# Citric Acid Changes the Fingerprint of Flavonoids and Promotes Their Accumulation in *Phellinus igniarius* (L.) Quél

**DOI:** 10.3390/life13010068

**Published:** 2022-12-26

**Authors:** Haoran Dong, Hui Chen, Bing Xu, Yingru Tan, Qun Ling, Liang Shi

**Affiliations:** Key Laboratory of Microbiological of Agricultural Environment, Ministry of Agriculture, College of Life Sciences, Nanjing Agricultural University, Nanjing 210095, China

**Keywords:** *Phellinus igniarius*, flavonoids, citric acid, fingerprint profiles

## Abstract

*Phellinus igniarius* is a valuable medicinal fungus. *P. igniarius* is rich in a variety of chemical compounds with medicinal value, among which are flavonoids. Therefore, increasing the content of flavonoids in *P. igniarius* is beneficial for its potential use in medicinal applications. This study demonstrated that exogenous treatment with citric acid (CA) could significantly increase flavonoid accumulation in *P. igniarius*. Additionally, we found that CA induced the biosynthesis of flavonoids in a concentration- and time-dependent manner. The flavonoid content could be increased up to 60.96 mg/g when using the treatment with 2.77 mM citric acid for 69.74 h, which was determined by using the response surface method. The changes in the fingerprint profiles of *P. igniarius* flavonoids with the treatment of CA as an exogenous inducer were also analyzed. In this study, the effect of citric acid as the exogenous inducer on the flavonoid content of *P. igniarius* was studied, and the processing conditions were optimized through the surface response curve. This approach provides novel insights and a theoretical basis for the production of high-quality *P. igniarius*.

## 1. Introduction

*Phellinus igniarius* is a valuable medicinal fungus, which belongs to the family Hymenochaetaceae (Basidiomycetes). It is known as the “gold in the forest” and has potential use in medicinal applications. In folk medicine, several species of Phellinus improve health and prevent or treat various diseases, such as alleviating oral ulcers, some gastroenteric disorders, and some lymphatic diseases. In the last decade, some Phellinus spp. have demonstrated additional medicinal properties, including anti-inflammatory, antidiabetic, antioxidant, immune-enhancing, antimutagenic, anticarcinogenic, and antimetastatic effects [1]. Modern pharmaceutical research has found that the fungus *P. igniarius* is rich in a variety of chemical compounds with medicinal value, such as flavonoids, polysaccharides, phenolic compounds, proteoglycans, triterpenes, and sterols [2,3,4]. Although it does not have the highest content of flavonoids, these compounds have strong biological activity and clear metabolic pathways. Flavonoids are considered one of the main medicinal active substances of Phellinus, and their effects include the inhibition of tumor growth, the removal of active oxygen, and reduced blood sugar, in addition to having anti-inflammatory and antiviral properties [4,5,6]. However, because of the undeveloped artificial cultivation, commercial products from this fungus are mostly obtained through field collection. Furthermore, due to the volatile environmental conditions and slow growth rate, the yield and quality of *P. igniarius* is unstable and significantly affect the cost of commercial products. Therefore, because of having a shorter processing time and fewer seasonal restrictions, submerged fermentation is known as a promising alternative for the efficient production of flavonoids from *P. igniarius*.

Previous studies have usually focused on increased flavonoid production through improvement in the extraction process or culture conditions. The optimization of culture conditions can increase the yield of flavonoids. Taking flavone content as the response value, the optimal medium formula was found as 3% wheat bran, 12% corn silk, 0.5% yeast extract, 0.15% KH_2_PO_4_, and 0.05% MgSO_4_ [7]. Zhu et al. revealed that the Vitreoscilla hemoglobin gene was expressed through chromosomal integration in Phellinus igniarius to alleviate oxygen limitation, and it increased the production of total flavones and exopolysaccharides 1.78- and 1.33-fold, respectively [8]. Zhang et al. showed that the use of stimulatory agents such as fatty acids, organic solvents, and surfactants can enhance the production of bioactive fungal exopolysaccharides from the edible mushroom Pleurotus tuber-regium [9]. Furthermore, organic acids can affect the secondary metabolism of the fungus by partially penetrating the fungal cell membrane to change its absorption capacity to the medium level. Ren et al. showed that AA can increase the content of ganoderic acids, an important secondary metabolite in *Ganoderma lucidum* [10]. Shi et al. found that salicylic acid induces flavonoid biosynthesis in a concentration- and time-dependent manner [11]. Wang found that citric acid (CA) can affect the metabolism of citrinin in *Monascus* [12]. A key intermediate in the tricarboxylic acid cycle is the CA, which is formed as a result of the condensation of acetyl CoA and oxaloacetate [13]. CA treatment stimulated the activities of the enzymes phenylalanine ammonia-lyase (PAL), cinnamate 4-hydroxylase (C4H), and 4-coumarin coenzyme A ligase (4CL) in the metabolism of phenylpropanoids and further accelerated the accumulation of flavonoid accumulation [14]. However, there is a paucity of reports as to whether CA affects the flavonoid content of *P. igniarius*. An increase in flavonoid content will increase the major bioactive component of *P. igniarius*, which enhances its nutritional value, in particular its therapeutic potential. The biological features of flavonoids in *P. igniarius* contribute to lowering blood pressure, treatment of cardiovascular diseases, and enhanced immunity, in addition to having anticancer and antitumor activity [1].

Additionally, flavonoids are a general term for a class of compounds that comprise a variety of different potent flavonoids, such as rutin, narcissoside, isoquercitrin, etc. Different flavonoid fractions have different pharmacological effects and targets. In a previous study, Xingtai Li et al. investigated the antioxidant effect of rutin and revealed its better inhibitory effect on lipid peroxidation and thus its protective effect on mitochondria [15]. Narcissoside has antiviral and immunity-boosting effects, whereas isoquercitrin has cough expectorant, hypotensive, antidepressant, anticancer, and hypoglycemic effects [16,17]. Increasing the content of different species of flavonoids helps to improve their therapeutic potential [18]. Nevertheless, it is also important to further enhance the different components.

In this study, the effects of CA on the accumulation and compound ratio of flavonoids in *P. igniarius* were investigated. The optimal induction conditions of citric acid were explored using the response surface methodology (RSM) with a central composite design (CCD), based on the effects of concentration and the treatment time of organic acids acting on flavonoids. The changes in the fingerprint profiles of *P. igniarius* flavonoids under the treatment of CA as an exogenous inducer were further analyzed.

## 2. Materials and Methods

### 2.1. Strain and Culture Conditions

*P. igniarius* (NACC6220) was provided by the Jiangsu Agricultural Microbial Germplasm Resources Collection of China. The fermentation experiments were performed in 250 mL flasks containing 100 mL of complete yeast medium (CYM) broth (1% maltose (biochemical grade, Sangon, Shanghai, China), 2% glucose (biotech grade, Sangon, Shanghai, China), 0.2% yeast extract (microbiological grade, Sangon, Shanghai, China), 0.2% peptone (microbiological grade, Sangon, Shanghai, China), 0.05% MgSO_4_·7H_2_O (biochemical grade, Sangon, Shanghai, China), and K_2_HPO_4_ (biochemical grade, Sangon, Shanghai, China) at 28 °C and 150 r·min^−1^ for 8 days.

### 2.2. Determination of Mycelial Biomass

The mycelia of *P. igniarius* were poured into a filter, and the culture solution was rinsed with water; then, the mycelia were spread on non-woven fabric, marked, and put on a drying rack in an oven (DGG-9053A, SUMSUNG, Shanghai, China) at 60 °C to dry to a uniform weight and then were weighed.

### 2.3. Determination of Flavonoid Content

Flavonoid content was measured using a microplate colorimetric assay based on the NaNO_2_-Al(NO_3_)_3_-NaOH-based colorimetric assay [19]. Briefly, 0.05 g of mycelium powder was placed on a plate and accurately weighed. Then, 70% ethanol was added under 1 h of ultrasound (KH-200KDB, HECHUANG ULTRASONIC, Jiangsu, China). Then, 1 mL supernatants were added with pipettes, and the solution was mixed with 0.2 mL of 5% NaNO_2_ (reagent grade, Sangon, Shanghai, China) and allowed to stand for 6 min in the dark at room temperature. Then, 0.2 mL 10% Al(NO_3_)_3_ (reagent grade, Sangon, Shanghai, China) was added, and the mixture was incubated at room temperature for 6 min. Afterward, 2 mL of 4% NaOH (reagent grade, Sangon, Shanghai, China) and 70% ethanol were added in order, shaken gently, and left to stand for 10 min. The absorbance was determined using a spectrophotometer (722, JINGHUA, Shanghai, China) at a wavelength of 510 nm.

### 2.4. Experimental Parameters of Surface Response Method

According to the single-factor test of citric acid (GHTECH, Guangdong, China), the concentration of citric acid and treatment time both have effects on the flavonoids of *P. igniarius*. Based on concentration and time, the surface response method was used to determine the optimal conditions of citric acid treatment. In this experiment, there were two test factors, namely concentration and time, so a central composite design (CCD) was adopted. Flavonoid content was the dependent variable (Y), while the treatment time and concentration of citric acid were the independent variables (X). The generalized form of the second-order polynomial equation is as follows:

(1) $$Y=\beta_{k0}+\sum_{i=1}^{5}\beta_{\mathrm{ki}}X_{i}+\sum_{i=1}^{5}\beta_{\mathrm{kii}}X_{i}^{2}+\sum_{i=1}^{4}\sum_{j=i+1}^{5}\beta_{\mathrm{kij}}X_{i}X_{j}$$

where *Y* is the response value of flavonoid content, *β*_k0_ is the intercept, *β*_ki_ is the linear effect factor, *β*_kii_ is the quadratic effect factor, and *β*_kij_ is the coefficient of interaction between the factors. The experimental data were analyzed using Design Expert 8.0 software.

### 2.5. Sample Preparations

Briefly, 0.2 g of mycelium powder treated with citric acid was weighed and put into a 10 mL centrifuge tube; then, 4 mL of 70% ethanol was added with a pipette gun, and the mixture was put into an ultrasound instrument for 2 h and centrifuged (HEXI, Hunan, China) for 10 min at a speed of 5000 r/min after the ultrasound. After centrifugation, 1 mL of the supernatant was drawn into a new 10 mL centrifuge tube and filtered in a vacuum dryer (DZF-6020, JINGHONG, Shanghai, China) until the liquid was completely dried. The liquid was completely evaporated. The remaining liquid was then added and drained, and after the liquid was completely drained, 600 µL of methanol was added to dissolve the liquid and sonicated in an ultrasonicator for 30–60 min. The liquid was then filtered through an organic filter membrane (0.22 µm).

For the chromatography of the standard product mixtures, 2 mg portions of quercitrin, rutin, narcissoside, kaempferol-3-O-rutinoside, quercetin, and sakura were weighed, dissolved in methanol, and filtered through an organic filter membrane (0.22 µm).

A standard addition experiment was performed for sample preparation. Three portions of 0.2 g *P. igniarius* mycelium were weighed and treated with the above method. Each aliquot was then split into two, thus resulting in six portions, three of which were injected and analyzed using a chromatographic instrument (LC-2030 Plus, SHIMADZU, Kyoto, Japan), and the other three were added to the control standards of 80%, 100%, and 120%, yielding six flavonoid samples.

### 2.6. Chromatographic Conditions

Column: Shim-pack GIST C18 5 µm (4.6I.D. × 250 mm). Mobile phase: A: 0.4% acetic acid (GHTECH, Guangdong, China) solution, B: methanol. Elution procedure: 0–4 min, 40%B; 4–9 min, 40–55%B; 9–30 min; 55%B; 30–40 min, 55%–70%B; 40–50 min, 70%B; 50–60 min, 70%–40%B; flow rate: 1 mL/min, detection wavelength: 362 nm, column temperature: 30 °C, injection volume: 5 µL.

### 2.7. Statistical Analysis

Three independent replicates of each set of experimental results were performed, and the average of the three sets was used as the final result of the data. The experimental results were achieved through independent analysis or multiple analyses [9].

## 3. Results

### 3.1. Effects of Different Concentrations of Citric Acid (CA) on Mycelial Biomass and Flavonoid Content of P. igniarius

**Figure 1:**
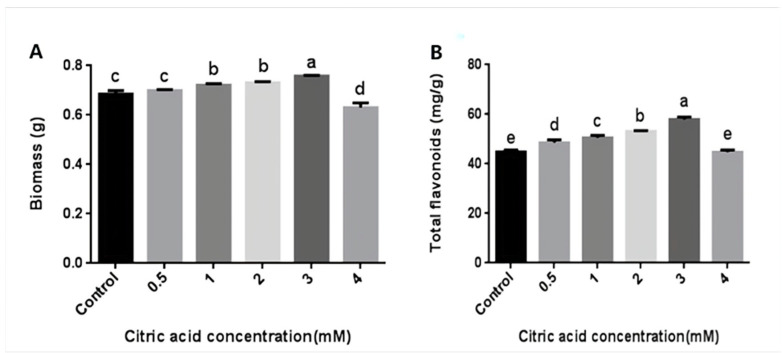
Effects of different concentrations of citric acid on mycelial biomass and total flavonoid content of *P. igniarius*: (**A**) effect of different concentrations of citric acid on mycelial biomass of *P. igniarius*; (**B**) effect of different concentrations of citric acid on total flavonoids of *P. igniarius* mycelia. Control sample was prepared without adding citric acid. The a, b, c, d, and e labels in the figure are the results of using different multiple-range tests to express the significance of difference. As shown in Figure 1A, there was no significant difference between control and 0.5 mM, 1 mM, and 2 mM concentrations, while there was a significant difference between the other two; as shown in Figure 1B, there was no significant difference between control and 4 mM concentration, while there was a significant difference between the other two. Each pair of experiments was repeated three times. All results are expressed in mean ± standard deviation. Different English letters indicate significant differences among different treatments (*p <* 0.05).

In order to explore whether CA could increase the content of flavonoids in the mycelia of *P. igniarius*, different concentrations of CA were added on the third day of fermentation. On the seventh day, the mycelia were harvested. The concentrations of CA treatment ranged from 0.5 mM (50 µL 1 mol/L CA) to 3 mM (300 µL 1 mol/L CA), and the mycelial biomass and flavonoid content showed an upward trend. As shown in Figure 1, when CA was added at 0.5 mM, there was no significant effect on the mycelial biomass (Figure 1A), but the content of flavonoids was increased. When the treatment concentration was 3 mM, the mycelial biomass and flavonoid content reached their maximum values of 0.758 g and 57.89 mg/g, respectively. Compared with the control group, the mycelial biomass and flavonoid content increased by 10.6% and 29.1%, respectively. When the treatment concentration was 4 mM (400 µL 1 mol/L CA), the mycelial biomass and flavonoid content were both lower than the control group, indicating that high concentrations of CA inhibited the growth of *P. igniarius* mycelia (Figure 1). These results indicate that an appropriate concentration of CA treatment can increase the mycelial biomass and flavonoid content of *P. igniarius*.

### 3.2. Effect of CA Treatment at Different Times on Mycelial Biomass and Flavonoid Content of P. igniarius

**Figure 2:**
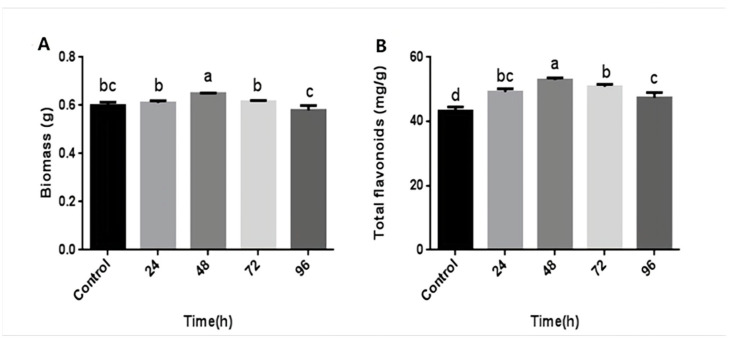
Effects of different times of citric treatment on mycelial biomass and total flavonoid content of *P. igniarius*: (**A**) effect of different times of citric acid treatment on mycelial biomass of *P. igniarius*; (**B**) effect of different times of citric acid treatment on total flavonoids of *P. igniarius* mycelia. Each pair of experiments was repeated three times. All results are expressed in mean ± standard deviation. Different English letters indicate significant differences among different treatments (*p <* 0.05).

The above results revealed that the mycelial biomass and flavonoid content of *P. igniarius* were highest when treated with CA at a concentration of 3 mM. Furthermore, hyphae were induced with 3 mM CA at different time points during fermentation to investigate the time effect. The results are shown in Figure 2. After 24–48 h of CA treatment, the mycelial biomass and flavonoid content both showed an upward trend. After 48 h of CA treatment, the mycelial biomass reached its maximum value, 0.648 g, which was an increase of 8.9%, compared with 0.595 g of the control group. After 72 h of CA treatment, the biomass showed a downward trend (Figure 2A). CA treatment time was in the range of 24–48 h. As the treatment time increased, the flavonoid content also increased. At 48 h, the flavonoid content reached its maximum value, 52.91 mg/g, which was 22.4% more than the control group. After 72–96 h of treatment, the content of flavonoids began to decrease (Figure 2B). These results indicate that CA treatment time also affects the mycelial biomass and flavonoid content of *P. igniarius*; a considerably long or short treatment time is not conducive to mycelial growth and flavonoid synthesis.

### 3.3. Surface Response Method to Optimize CA Treatment Conditions

The above results showed that the concentration and time point of CA treatment affected the flavonoid content of *P. igniarius* mycelia. Based on the results of our single-factor experiments, a five-level two-factor test was carried out using the central composite design (CCD) method using Design-Expert 8.0 software. In total, 21 combinations were obtained, as shown in Table 1. These 21 combinations of CA treatment time and concentration were used to perform the fermentation test again to obtain the flavonoid content values. Then, using Design-Expert 8.0 software, the data were analyzed, the results of which are shown in different tables. The CA CCD test design is shown in Table 1, the response surface model analysis is shown in Table 2, and the regression analysis is shown in Table 3. The quadratic multiple regression equation was obtained after the software analysis of the data in Table 1 as follows:

Y = 46.59 + 0.39 × Time + 9.50 × Concentration + 9.66 × 10^−3^ × Time × Concentration 4.04 × 10^−3^ × Time^2^ − 2.54 × Concentration^2^.

In this equation, Y indicates the content of flavonoids and is used as the dependent variable, while Time and Concentration are the time and concentration of CA treatment. According to Table 2, the F-value of the model was 66.39, the *p*-value (Prob > F) was less than 0.0001, and the F-value of lack of fit was 20.37. These data indicate that the model built with the software yielded statistically significant results. The correlation coefficient of R^2^ = 0.9094 was obtained, which is greater than 0.8, indicating a good degree of fit between the actual value of the flavonoid content and its predicted value obtained with software analysis.

**Figure 3:**
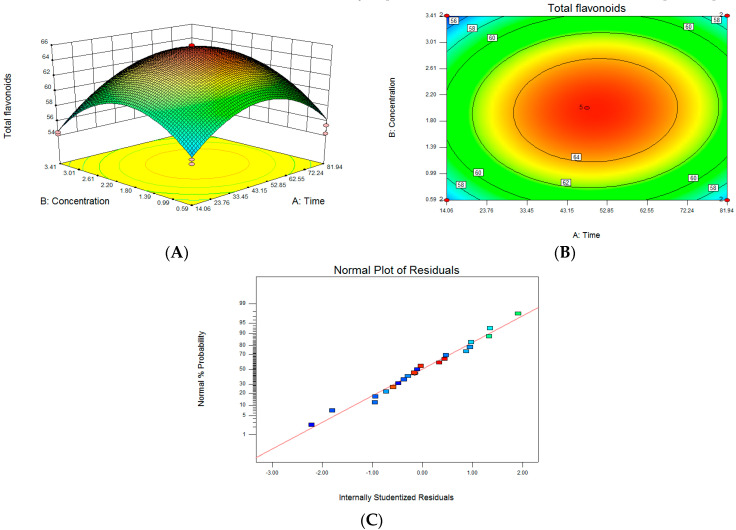
Optimization of treatment conditions for citric acid: (**A**) citric acid treatment time and concentration interaction response surface model; (**B**) the contour of two-factor interaction; (**C**) the normal probability plot of the standardized residuals.

Figure 3A illustrates the response surface results of the interaction between CA treatment time and concentration. It can be seen that the Z axis has the maximum value. As shown in Figure 3C, each point is almost on a straight line, indicating that the analysis result is reliable. According to the software analysis and optimization results, the concentration of CA treatment was 2.77 mM (277 µL 1 mol/L CA), the treatment time of lemon was 69.74 h, and the predicted value of Y in our software analysis was 62.55 mg/g.

In terms of the optimized results, the CA treatment concentration was 2.77 mM, the treatment time was 69.74 h, and the CA treatment liquid fermentation test was re-run. The three parallel test results were 62.87 mg/g, 60.91 mg/g, and 58.99 mg/g. The average value was 60.96 mg/g, which was not much different from the predicted value of 62.55 mg/g, indicating that the optimization of CA treatment conditions using the surface response method achieved statistically significant results and can be used as a guiding reference.

### 3.4. Effects of CA Treatment on Flavonoid Fingerprint of P. igniarius Mycelia

**Figure 4:**
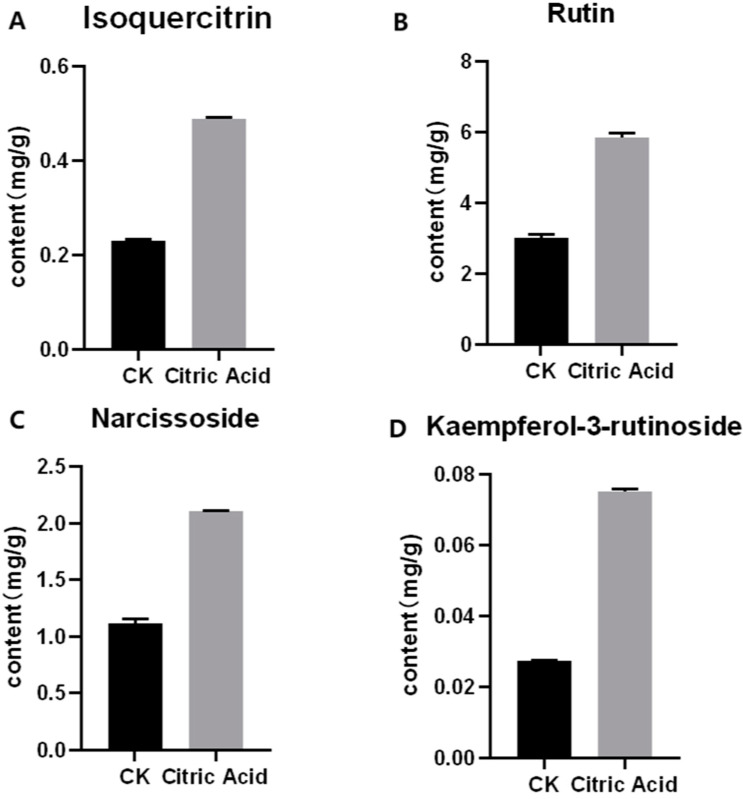
Effect of citric acid treatment on the content of four flavonoids: (**A**) effect of citric acid treatment on the content of isoquercitrin; (**B**) effect of citric acid treatment on the content of rutin; (**C**) effect of citric acid treatment on the content of narcissoside; (**D**) effect of citric acid treatment on content of kaempferol-3-rutinoside. Each treatment was repeated three times. All results are expressed as mean ± standard deviation.

**Figure 5:**
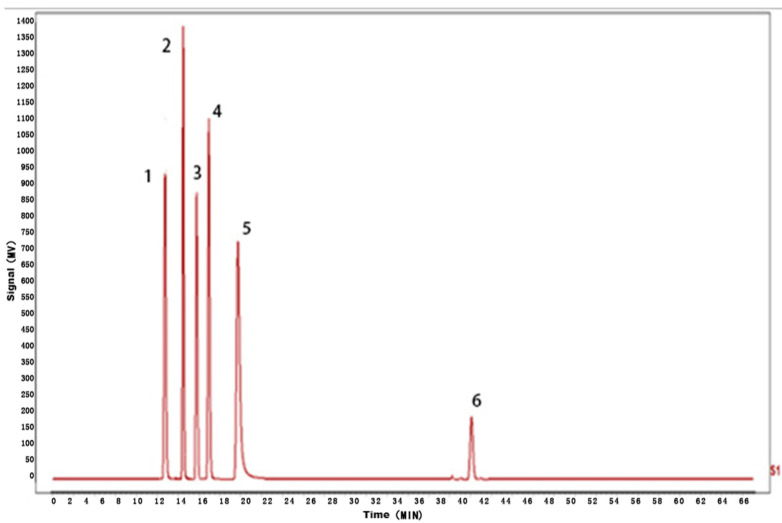
HPLC fingerprint of mixed markers.

**Figure 6:**
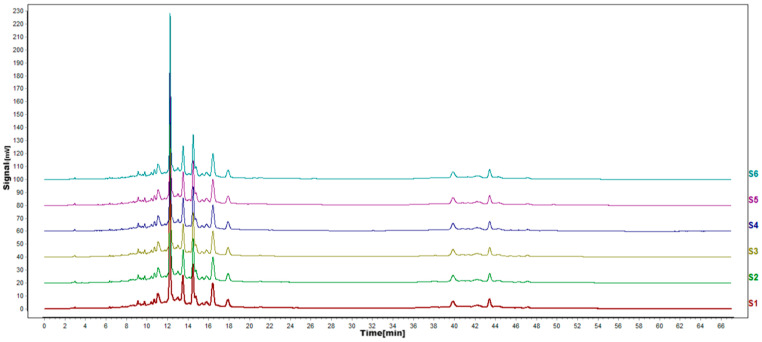
HPLC fingerprints of accuracy test. S1–S6 in the map are the fingerprints of the same sample solution of *P. igniarius* that was sampled 6 consecutive times.

**Figure 7:**
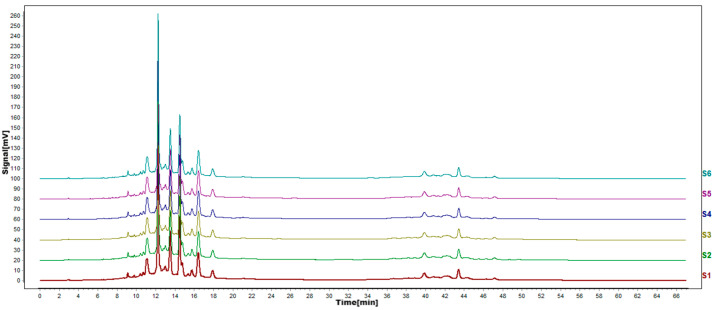
HPLC fingerprints of renaturation test. S1–S6 are the flavonoid HPLC fingerprints of *P. igniarius* mycelium of the same batch.

**Figure 8:**
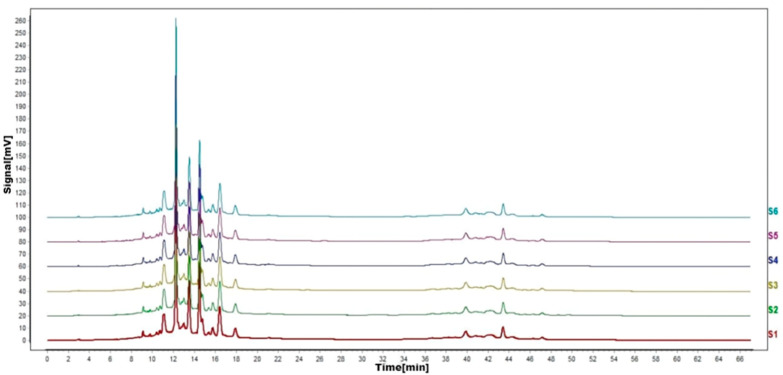
HPLC fingerprints of stability test. S1–S6 are flavonoid HPLC fingerprints of the same test solution at different times.

**Figure 9:**
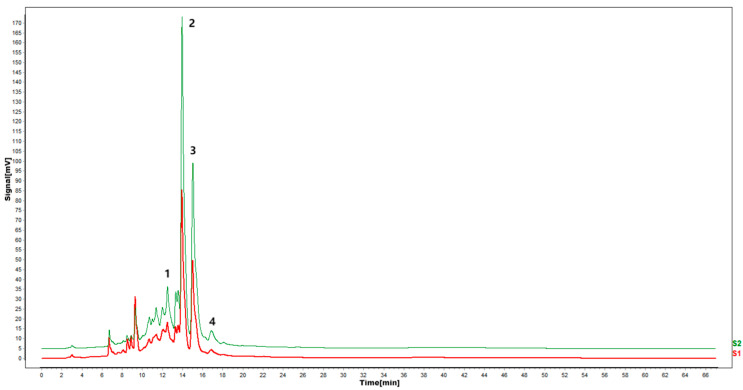
Effect of citric acid treatment on flavonoid fingerprints of *P. igniarius* mycelia. S1 is the flavonoid HPLC fingerprint of *Phellinus igniarius* mycelium of control, and S2 is the flavonoid HPLC fingerprint of *Phellinus* mycelium with citric acid treatment. Each treatment was repeated three times: 1—isoquercitrin; 2—rutin; 3—narcissoside; 4—kaempferol-3-rutinoside.

According to the results of HPLC analysis, CA treatment could increase the flavonoid content of *P. igniarius* Mycelia. However, flavonoids are a general term for a variety of compounds. Although CA treatment could increase the total flavonoid content, it was still unclear whether it affected the content of flavonoids with important activities. In order to further analyze the effects of CA on the content of the herbal compounds in flavonoids, the fingerprint of each group was analyzed via HPLC. According to the above method, six flavonoids were obtained, namely isoquercetin, rutin, narcissoside, kaempferol-3-O-rutinoside, quercetin, sakuranetin, and the regression equation is shown in Table 4. The changes in the content of flavonoids of the CA-treated group with important medicinal activity are shown in Figure 4. S1 is the HPLC fingerprint of *P. igniarius* flavonoids in the control group, and S2 is the HPLC fingerprint of *P. igniarius* flavonoids treated with CA. According to the peak times of the six flavonoids and the HPLC chromatogram of the six flavonoid standard mixtures (Figure 5), 1—isoquercitrin, 2—rutin, and 3—narcissoside, 4—kaempferol-3-O-rutinoside, 5—quercetin, and 6—sakuranetin. This result was analyzed with an accuracy test (Figure 6), renaturation test (Figure 7), stability test (Figure 8), and spike experiments (Table 5). At the same time, CA treatment also showed different degrees of increase in the first four compounds. As shown in Figure 9, after CA treatment, the content of isoquercitrin increased by 111.67%, compared with the control group; the content of rutin increased by 94.85%, compared with the control group; the content of narcissoside increased by 88.97%, compared with the control group; and kaempferol-3-O-rutinoside increased by 178.76%, compared with the control group. This result shows that CA treatment can increase the content of four important active flavonoids in *P. igniarius* mycelium.

## 4. Discussion

*P. igniarius* is a valuable medicinal fungus. Flavonoids are the main active substances in *P. igniarius*. They have antibacterial and anti-inflammatory properties and contribute to the inhibition of virus reproduction and tumor growth and removal of active oxygen [20]. However, their concentration is relatively low, which seriously limits the development of *P. igniarius* in food products and its economic value. In this article, to improve the content of flavonoids in *P. igniarius*, the effect of exogenous inducers on the flavonoid content of *P. igniarius* was investigated. The optimum conditions for the treatment of *P. igniarius* mycelia were found using the surface response method, and the effects of CA on the biomass and flavonoid content of *P. igniarius* were explored. According to the analysis results of HPLC fingerprints, CA could induce an increase in the content of different flavonoids. It is well known that CA is a key intermediate in the tricarboxylic acid cycle and is formed by the condensation of acetyl CoA and oxaloacetate. Moreover, the process of flavonoid synthesis in *P. igniarius* is yet to be studied: The biosynthesis of flavonoids starts with phenylalanine, which is catalyzed by phenylalanine ammonia-lyase (PAL), cinnamate 4-hydroxylase (C4H), and 4-coumarin coenzyme A ligase (4CL), the core biosynthetic genes related to phenylalanine, and provides precursors for the biosynthesis of the main phenolic secondary metabolites of *P. igniarius* [21,22]. CA treatment may stimulate PAL, C4H, and 4CL enzyme activities in phenylpropanoid metabolism and further accelerate the accumulation of flavonoids. However, the concentration of citric acid should be controlled. Higher concentration of citric acid will inhibit the activity of key enzymes, affecting energy supply and flavonoids synthesis of *P. igniarius*. Therefore, a high concentration of citric acid will inhibit the growth of *P. igniarius* [13,14].

In recent years, HPLC fingerprinting of traditional Chinese medicines has enabled a clear identification of specific active chemical compounds and the evaluation of their efficacy, which is of great significance to achieving high-quality standardization in traditional Chinese medicines. Li et al. used HPLC to accurately determine the content of seven kinds of flavonoids in plants of the genus *Ligustrum*, which helped to understand the changes in the content of flavonoids in different planting areas and, therefore, is conducive to the production of high-quality plants [23]. Zhao et al. used HPLC to determine the fingerprints of different origins and grades of Digu skin and accurately determined the content of important flavonoids, which provided a basis for quality control [24]. Li et al. analyzed the fingerprint of *Cockscomb* medicinal material using HPLC and determined the content of important flavonoids [25]. Exploring the changes in the fingerprint of flavonoids is of great significance for the evaluation of the efficacy of *P. igniarius* and the cultivation of high-quality flavonoids. The development and application of fingerprint mapping have great potential in solving the bottlenecks in the development of the mulberry industry and will further promote the research and development of fungi for food and medicinal applications.

Some studies claim that organic acids can increase the growth rate of the hyphae, biomass, and content of some secondary metabolites of edible fungi [26,27]. In this study, when the CA concentration was 3 mM, the mycelial biomass and flavonoid content were the highest. Regarding the time of single-factor treatment, the biomass and flavonoid content reached their maximum at 48 h, and these results are similar to those reported by Zhang et al. [9]. The effects were concentration- and time-dependent. The peak biomass was reached at a treatment time of 120 h and a concentration of 3 g/L. The results of Liu et al. showed that the traditional Chinese medicine red ginseng treated with CA has better anti-fatigue properties and regulates immunity better than untreated varieties due to significant improvement in T-SOD, IL-2, and IgG and a decrease in MDA [28]. To increase the biomass content of *P. igniarius*, naphthalene AA and oleic acid were used as exogenous inducers by Zhu et al. The authors screened the optimal compounds of media and significantly increased the mass of polysaccharides, causing them to reach the maximum values at concentrations of 0.2 mg/L and 1 g/L, respectively [29]. Aside from changing the culture medium, culture conditions, growth environment, and other factors, the addition of exogenous inducers can increase the content of flavonoids and other secondary metabolites. Organic acids can increase the biomass and secondary metabolite content of mycelia, relative to the increase in the absorbed nutrients. It has been established that organic acids can increase the uptake of nutrients by partially entering the fungal cell membrane. There are also reports that the appropriate organic acid concentration can stimulate microorganisms to use carbon sources, thus increasing the content of metabolites [30]. Exogenous CA treatment increased the flavonoid content of *P. igniarius*, as verified in this paper, and the changes in their flavonoid content may be caused by changes in the metabolic levels induced by organic acids. The safe and efficient increase in the flavonoid content of important medicinal compounds in *P. igniarius* by using cheap organic acids can greatly improve the quality of *P. igniarius* and promote its development.

Flavonoids are a general term for a large group of compounds, and different flavonoids have different medicinal functions. By changing the content of different flavonoids, the biological functions of flavonoids can be changed, and other traditional medicinal materials can also be changed in the same way. Increasing the content of flavonoids increases the main bioactive component of *P. igniarius*, which improves its nutritional value, especially its medicinal potential. Studies show that different flavonoids have different biological activities. Rutin regulates the endocrine system, protects the cardiovascular system, promotes digestion, and has anticancer and antitumor properties [31]. Isoquercitrin has high bioavailability and exerts several chemoprotective effects, both in vitro and in vivo, against oxidative stress, cancer, cardiovascular disorders, diabetes, and allergic reactions [32]. Narcissoside has antioxidant, anticholinergic, antidiabetic, anti-acute myeloid leukemia, and antiviral effects [33,34]. Kaempferol-3-O-rutinoside has anti-inflammatory, anti-saccharification, and hypotensive effects [16,35,36]. Understanding the changes in the fingerprint of flavonoids is of great significance for the evaluation of the efficacy of *P. igniarius* and the cultivation of high-quality flavonoids. In traditional Chinese medicine, it is very common to improve biological activity by changing the content of flavonoids. The results of a study by Xie, Zhuohong showed that the content of flavonoids in the diploid Gynostemma pentaphyllum was significantly higher than that of tetraploid Gynostemma pentaphyllum, especially rutin and quercetin, which improved the antioxidant, antiproliferative, and anti-inflammatory activities [37]. According to Wang et al., under low salt stress, it is more conducive for licorice to accumulate flavonoids, triterpenoids, and other bioactive substances [38]. The difference in the content of different flavonoids will affect the medicinal and nutritional value of traditional Chinese medicine. Rutin, isoquercetin, and kaempferol-3-O-rutinoside all have the effects of protecting the cardiovascular system and lowering blood pressure and, therefore, can be used for the development of antihypertensive drugs. Rutin and isoquercetin have anticancer and antitumor effects and can be used as anticancer drugs. Narcissoside is a potent inhibitor of viral COVID-19 protein 6W63 and was pharmacologically and clinically evaluated for the treatment of the novel coronavirus disease 2019 (COVID-19) [36]. In this study, high-performance liquid chromatography (HPLC) was used to analyze the changes in the fingerprint of flavonoids in mycelia treated with CA. The contents of isoquercitrin, rutin, narcissoside, and kaempferol-3-O rutinoside were found to be increased to different degrees in the organic acid (CA)-inducer-treated seeds compared with the control group. CA revealed a better effect on increasing the content of kaempferol-3-O rutinoside, as its content increased by 178.76%, compared with the control group. Isoquercitrin increased by 111.67%, second only to kaempferol-3-O rutinoside. Therefore, through the induction of CA, the potential of *P. igniarius* in lowering blood pressure, treating cardiovascular diseases, and enhancing immunity, as well as its anticancer and antitumor effects, was significantly improved. However, we also found that other peaks also increased, and we speculate that the biological activity of flavonoids would also change. Therefore, in future studies, we plan to further explore the changes in these substances and the improvement in their biological activities.

## 5. Conclusions

The induction of CA increased mycelial biomass and flavonoid content in *P. igniarius*, which is of great importance to *P. igniarius* production and application. In addition, the differences in various flavonoid compounds help to significantly enhance the medicinal potential of *P. igniarius* and, therefore, are beneficial in providing methods for the further development of products with different biological activities.

## Figures and Tables

**Table 1:** Design matrix of citric acid CCD.

Run	Factor 1	Factor 2	Response 1
	A: Time (h)	B: Concentration (mM)	Flavonoid (mg/g)
1	48.00	0.00	56.65 ± 3.76
2	14.00	3.41	54.45 ± 1.19
3	48.00	4.00	55.89 ± 2.31
4	48.00	0.00	56.98 ± 4.54
5	0.00	2.00	56.21 ± 1.48
6	14.00	0.59	55.17 ± 1.99
7	82.00	3.41	55.04 ± 3.26
8	48.00	4.00	55.47 ± 2.30
9	82.00	0.59	55.38 ± 1.20
10	0.00	2.00	55.12 ± 2.72
11	48.00	2.00	65.89 ± 3.29
12	48.00	2.00	65.42 ± 3.85
13	82.00	3.41	55.98 ± 1.01
14	82.00	0.59	54.27 ± 2.86
15	14.00	3.41	54.12 ± 2.80
16	48.00	2.00	65.54 ± 1.57
17	96.00	2.00	58.23 ± 1.81
18	48.00	2.00	65.02 ± 1.65
19	48.00	2.00	65.99 ± 2.35
20	96.00	2.00	58.74 ± 4.67
21	14.00	0.59	55.74 ± 1.88

Note: Each experimental combination was performed in parallel three times, and the response value was the average of the three experimental results. R^2^ = 0.9094 > 0.8.

**Table 2:** ANOVA for response surface quadratic model.

Source	Sum of Squares	DF	Mean Squares	F-Value	*p*-Value (Prob > F)
Model	370.24	5	74.05	66.39	<0.0001
Residual	16.73	15	1.12		
Lack of fit	13.98	3	4.66	20.37	<0.0001
Pure error	2.75	12	0.23		
Cor total	386.97	20			

**Table 3:** The least-square fit and coefficient estimate.

Factor	Coefficient Estimate	Standard Error	%95CI Low	%95CI High	F-Value	*p*-Value (Prob > F)
Intercept	65.57	0.47	64.57	66.58		
A: Time	0.57	0.26	0.010	1.14	4.71	0.0465
B: Concentration	−0.26	0.26	−0.82	0.30	0.98	0.3381
AB	0.46	0.37	−0.33	1.26	1.54	0.2333
A^2^	−4.66	0.33	−5.36	−3.96	200.91	<0.0001
B^2^	−5.07	0.33	−5.77	−4.37	238.18	<0.0001

**Table 4:** Regression equation.

Compound	Regression Equation	R^2^	Linear Rage (mg/mL)
Isoquercitrin	Y = 10^7^X + 20,031	0.9998	0.00625–0.1
Rutin	Y = 3 × 10^6^X + 63,753	0.9995	0.1–2
Narcissoside	Y = 7 × 10^6^X − 11,287	0.9994	0.025–0.5
Kaempferol-3-rutinoside	Y = 10^7^X + 12,755	0.9994	0.0125–0.2
Quercetin	Y = 5 × 10^6^X + 44,515	0.9961	0.00625–0.2
Sakuranetin	Y = 10^6^X − 813.67	0.9988	0.0125–0.2

**Table 5:** Spiked experiments.

Compound	Original Content (mg)	Addition (mg)	Total Content (mg)	Return (%)	Mean Recovery (%)	RSD (%)
Isoquercitrin	0.152	0.122	0.270	98.54	97.24	1.41
	0.152	0.152	0.296	97.37		
	0.152	0.182	0.320	95.81		
Rutin	1.142	0.914	1.990	96.80	6.90	1.84
	1.142	1.142	2.174	95.18		
	1.142	1.370	2.480	98.73		
Narcissoside	0.308	0.246	0.544	98.19	97.46	1.15
	0.308	0.308	0.596	98.03		
	0.308	0.370	0.652	96.17		
Kaempferol-3-rutinoside	0.104	0.084	0.180	95.74	97.04	1.30
	0.104	0.104	0.202	97.12		
	0.104	0.124	0.224	98.25		
Quercetin	0.096	0.076	0.170	98.84	97.66	1.35
	0.096	0.096	0.188	97.92		
	0.096	0.116	0.204	96.23		
Sakuranetin	0.830	0.664	1.448	96.92	96.76	1.67
	0.830	0.830	1.632	98.31		
	0.830	0.996	1.736	95.07		

## Data Availability

The data that support the findings of this study are available from the corresponding author upon reasonable request.
